# Disrupting Smad3 potentiates immunostimulatory function of NK cells against lung carcinoma by promoting GM-CSF production

**DOI:** 10.1007/s00018-024-05290-4

**Published:** 2024-06-15

**Authors:** Guang-Yu Lian, Qing-Ming Wang, Thomas Shiu-Kwong Mak, Xiao-Ru Huang, Xue-Qing Yu, Hui-Yao Lan

**Affiliations:** 1 Guangdong-Hong Kong Joint Research Laboratory on Immunological and Genetic Kidney Diseases, Departments of Pathology and Nephrology, Guangdong Provincial People’s Hospital, Guangdong Academy of Medical Sciences, Guangzhou, 510080 China; 2 Guangdong Cardiovascular Institute, Guangdong Provincial People’s Hospital, Guangdong Academy of Medical Sciences, Guangzhou, 510080 China; 3 Department of Medicine & Therapeutics, and Li Ka Shing Institute of Health Sciences, The Chinese University of Hong Kong, Hong Kong SAR, China; 4 Department of Hematology, The Second Affiliated Hospital of Nanchang University, Nanchang, Jiangxi China

**Keywords:** TGF-β, NK cells, Neutrophils, Adoptive cell therapy, Immunoregulation, GM-CSF

## Abstract

Through Smad3-dependent signalings, transforming growth factor-β (TGF-β) suppresses the development, maturation, cytokine productions and cytolytic functions of NK cells in cancer. Silencing Smad3 remarkably restores the cytotoxicity of NK-92 against cancer in TGF-β-rich microenvironment, but its effects on the immunoregulatory functions of NK cells remain obscure. In this study, we identified Smad3 functioned as a transcriptional repressor for *CSF2* (GM-CSF) in NK cells. Therefore, disrupting Smad3 largely mitigated TGF-β-mediated suppression on GM-CSF production by NK cells. Furthermore, silencing GM-CSF in Smad3 knockout NK cells substantially impaired their anti-lung carcinoma effects. In-depth study demonstrated that NK-derived GM-CSF strengthened T cell immune responses by stimulating dendritic cell differentiation and M1 macrophage polarization. Meanwhile, NK-derived GM-CSF promoted the survival of neutrophils, which in turn facilitated the terminal maturation of NK cells, and subsequently boosted NK-cell mediated cytotoxicity against lung carcinoma. Thus, Smad3-silenced NK-92 (NK-92-S3KD) may serve as a promising immunoadjuvant therapy with clinical translational value given its robust cytotoxicity against malignant cells and immunostimulatory functions to reinforce the therapeutic effects of other immunotherapies.

**Supplementary Information:**

The online version contains supplementary material available at 10.1007/s00018-024-05290-4.

## Introduction

The revolutionary success achieved by CAR-T cell therapy against hematologic malignancies inspires the development of myriads novel adoptive cell therapies, among which NK cell-based therapy represents a promising candidate owing to its fast immune response, “off-the-shelf” feasibility, potent immune regulatory capacity and graft-versus‐leukemia (GVL) effects without causing graft versus host disease (GVHD) [1, 2]. Compared with T cell-based therapies, NK cell-based therapies circumvent limitations such as the dependence on antigen presentation and tumor antigen heterogeneity [3].

Despite significant advances in hematologic malignancies, NK cell-based therapeutic strategies suffered from numerous obstacles in solid cancers [4, 5]. Although combining with immune checkpoint blockade significantly improves the efficacy of NK cell-based therapies, the systemic immune checkpoint blockade therapy would also contribute to frequent immune-related adverse events (irAEs) [6]. Therefore, the development of novel strategy to alter a supportive tumor microenvironment to an anti-tumor one is in urgent need to promote the therapeutic efficacy of NK cell-based immunotherapies. TGF-β-induced immunosuppression substantially impairs NK cell-mediated cytotoxicity against cancer [7, 8]. High level TGF-β impedes the egression of immature NK cells from the bone marrow by increasing the expression of CXCR4 while reducing CX3CR1 [9, 10]. TGF-β also blunts the activation of NK cells by overexpressing the inhibitory receptors while suppressing the activating receptors, as well as by inducing ligand shedding, thus switching the stimulatory signalings to the inhibitory ones [11]. Furthermore, TGF-β largely diminishes CD16-mediated antibody-dependent cellular cytotoxicity (ADCC) and cytokine releasing capacity of NK cells [12]. Previous studies also identified Smad3, a key transcription factor in the canonical TGF-β signaling, as a transcriptional repressor for T-bet, E4BP4, Id2 and IRF2, which are essential transcriptional regulators for NK cell maturation, effector functions and interferon γ (IFN-γ) production [13–15]. Thus silencing Smad3 in NK cells remarkably increases IFN-γ production as well as the cytotoxicity against cancer [16].

Apart from killing tumor cells through perforin-dependent and death-receptor-dependent pathways, NK cells also orchestrate the immune microenvironment by producing cytokines and chemokines. For instance, as one of the major sources of IFN-γ, NK cells promote the activation of T helper (Th) cells, effector CD8^+^ T cells, dendritic cells (DCs), macrophages, neutrophils and even NK cells themselves in positive feedback pathways [17]. In this study, we explored the regulatory roles of Smad3 on cytokine secretion by NK cells with cytokines array. Interestingly, GM-CSF was found as the most significantly enhanced cytokine produced by Smad3-silencing NK-92 cells compared with control NK-92 cells.

GM-CSF functions as a double-edged sword in cancer. As a potent differentiation regulator, GM-CSF decelerates tumor progression by inducing terminal differentiation of cancer stem cells and cell cycle arrest at the G0/G1 phase in small cell lung cancer cell (SCLC) cell lines [18, 19]. While other studies found that GM-CSF facilitates tumor proliferation and metastasis by promoting the proteolytic functions of MMPs and uPA in an autocrine manner in non-small cell lung cancer (NSCLC) cell lines [20, 21]. GM-CSF also serves as a prognostic marker with outstanding sensitivity in early stage NSCLC patients [22]. More importantly, GM-CSF plays an essential role in immunoregulation in the tumor microenvironment. Through promoting the differentiation and maturation of DCs, GM-CSF facilitates CD8^+^ T cell priming and strengthens the cytokine productions by CD4^+^ T cells [23]. Meanwhile, GM-CSF triggers the differentiation of Flt3^+^ monocytes to inflammatory monocyte-derived dendritic cells (moDCs), which exercise potent anti-tumor activities through reactive oxygen species (ROS)-dependent mechanisms, as well as by priming T cells through PPARγ-dependent pathways [24–26]. GM-CSF also induces the pro-inflammatory polarization in Flt3^−^ monocytes, which subsequently contributes to the proliferation and activation of antigen specific CD8^+^ T cells through the production of inflammatory cytokines such as TNF-α and IL-1β [27–29]. However, GM-CSF induces severe immunosuppression by mediating the activation and recruitment of regulatory T (Treg) cells and myeloid-derived suppressor cells (MDSCs) [30, 31]. Given the versatile roles of GM-CSF in different types of cancer and immune cells, the exact functions of NK derived GM-CSF in lung carcinoma has not yet been elucidated. Thereby, in the present study, we explored the mechanisms underlying Smad3-mediated inhibition on GM-CSF production by NK cells as well as how NK-derived GM-CSF sculpted the immune microenvironment in lung carcinoma.

## Results

### Smad3 as a transcriptional repressor for CSF2 in NK cells

**Fig. 1:**
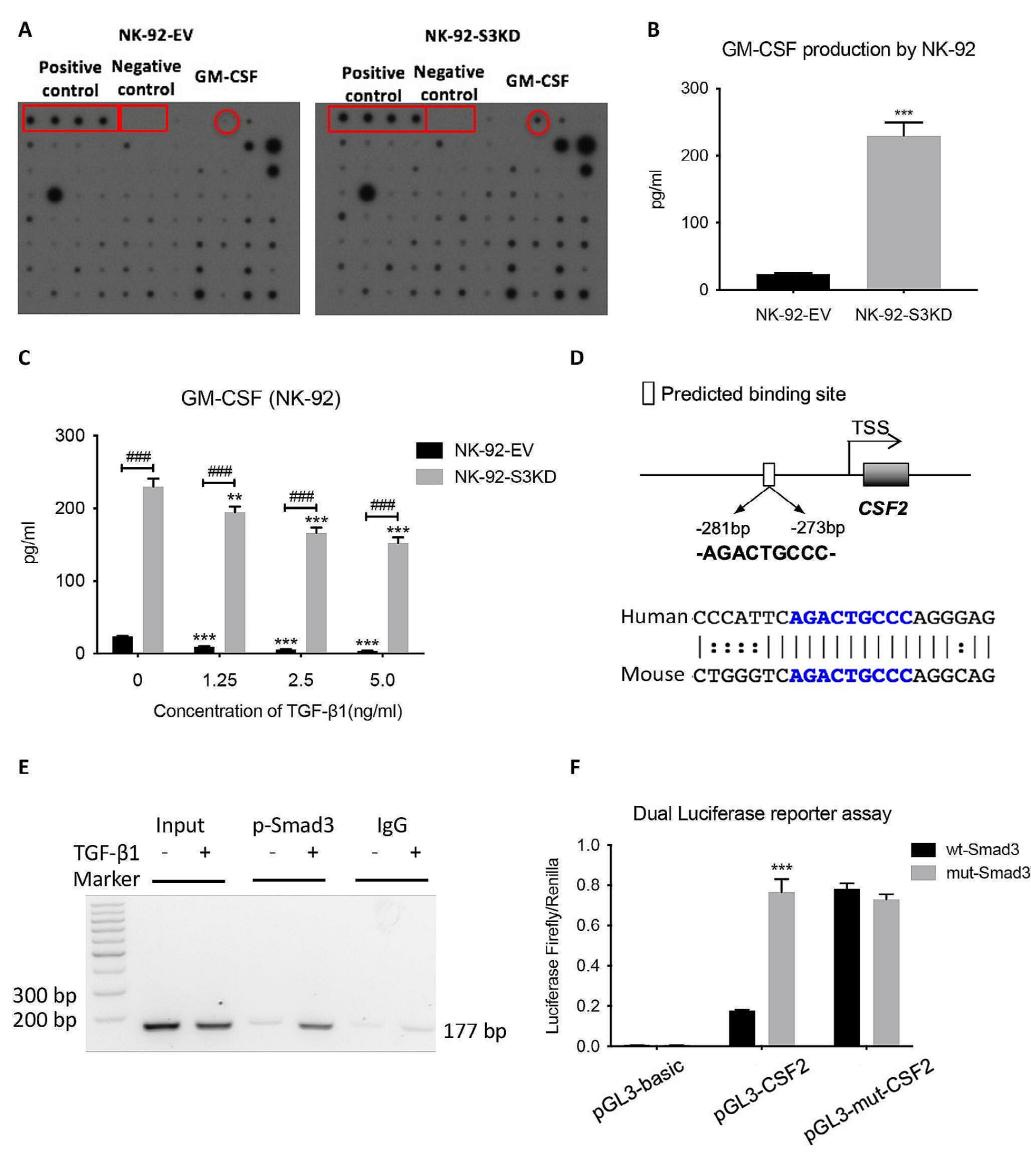
TGF-β suppresses GM-CSF production in NK cells through Smad3-mediated transcriptional regulation. **(A)** Cytokine profiling detecting 80 different cytokines or chemokines in NK-92-EV and NK-92-S3KD supernatant. **(B)** GM-CSF levels in the supernatant of NK-92-EV and NK-92-S3KD cells determined by ELISA. Each bar represents the mean ± SD for groups of three independent experiments. *** *p* < 0.001 compared to NK-92-EV. **(C)** ELISA determining GM-CSF levels in NK-92-EV and NK-92-S3KD cells stimulated with recombinant TGF-β1 for 24 h. Each bar represents the mean ± SD for groups of three independent experiments. ** *p* < 0.01, *** *p* < 0.001 compared to no TGF-β stimulation; ### *p* < 0.001 as indicated. **(D)** Schematics of predicted Smad binding site on *CSF2* gene. **(E)** ChIP assay verified the physical interaction between p-Smad3 and its predicted binding site in the promoter region of *CSF2* gene. **(F)** Dual luciferase reporter assay validated Smad3 functions as a transcriptional repressor for *CSF2* gene. wt-Smad3: pcDNA3.1 vector containing Smad3; mut-Smad3: pcDNA3.1 vector containing mutant Smad3. pGL3-basic: original pGL3 luciferase reporter basic vector; pGL3-CSF2: pGL3 vector containing *CSF2* promoter sequence; pGL3-mut-CSF2: pGL3 vector containing *CSF2* promoter sequence with a point mutation on the predicted Smad binding site. Each bar represents the mean ± SD for groups of three independent experiments. *** *p* < 0.001 compared to wt-Smad3

To explore the role of Smad3 in the cytokine and chemokine productions by NK cells, we firstly performed cytokine profiling with supernatant from empty vector (EV)-transfected NK-92 (NK-92-EV) cells and Smad3-silencing NK-92 (NK-92-S3KD) cells generated as described previously [16]. As shown in Fig. 1A, GM-CSF was the most remarkably changed cytokine among the 80 proteins in NK-92-S3KD, ten times higher than that in NK-92-EV supernatant. This indicated Smad3 may be a critical regulator for GM-CSF production in NK-92 cells. As Smad3 is one of the key transcription factors in canonical TGF-β signaling, we then evaluated the impact of TGF-β1 stimulation on the production of GM-CSF in NK-92 cells. Consistent with cytokine array results, silencing Smad3 substantially increased the level of GM-CSF produced by NK-92 cells (Fig. 1B-C). However, TGF-β1 stimulation inhibited the production of GM-CSF in both NK-92-EV and NK-92-S3KD (Fig. 1C), implying the participation of TGF-β-mediated Smad3-independent regulations.

Promoter analysis identified a predicted Smad binding site on the promoter region of *CSF2* (GM-CSF) conserved between mouse and human with Evolutionary Conserved Regions (ECR) browser (rVista 2.0, https://rvista.dcode.org/) and JASPAR database (http://jaspar.genereg.net) (Fig. 1D) [32, 33]. Chromatin immunoprecipitation (ChIP) assay was performed thereupon to verify this transcription regulation. As shown in Fig. 1E, TGF-β1 stimulation enhanced the binding of p-Smad3 to the predicted Smad binding site on the promoter of *CSF2*. Dual luciferase reporter assay was subsequently carried out to validate whether Smad3 regulates the transcription of *CSF2* through binding to the specific binding site. The transfection of a mutant Smad3 containing plasmid markedly promoted the transcription activity of the *CSF2* promoter compared with the transfection of a Smad3 containing plasmid, indicating the negative regulation of GM-CSF expression by Smad3. Meanwhile, the introduction of a point mutation on the predicted Smad binding site on the *CSF2* promoter also abrogated the suppression of Smad3 on the transcription activity of the *CSF2* promoter (Fig. 1F). Taken together, Smad3 acted as a transcriptional repressor in TGF-β1-mediated suppression on GM-CSF production.

### NK-derived GM-CSF suppresses lung carcinoma progression

**Fig. 2:**
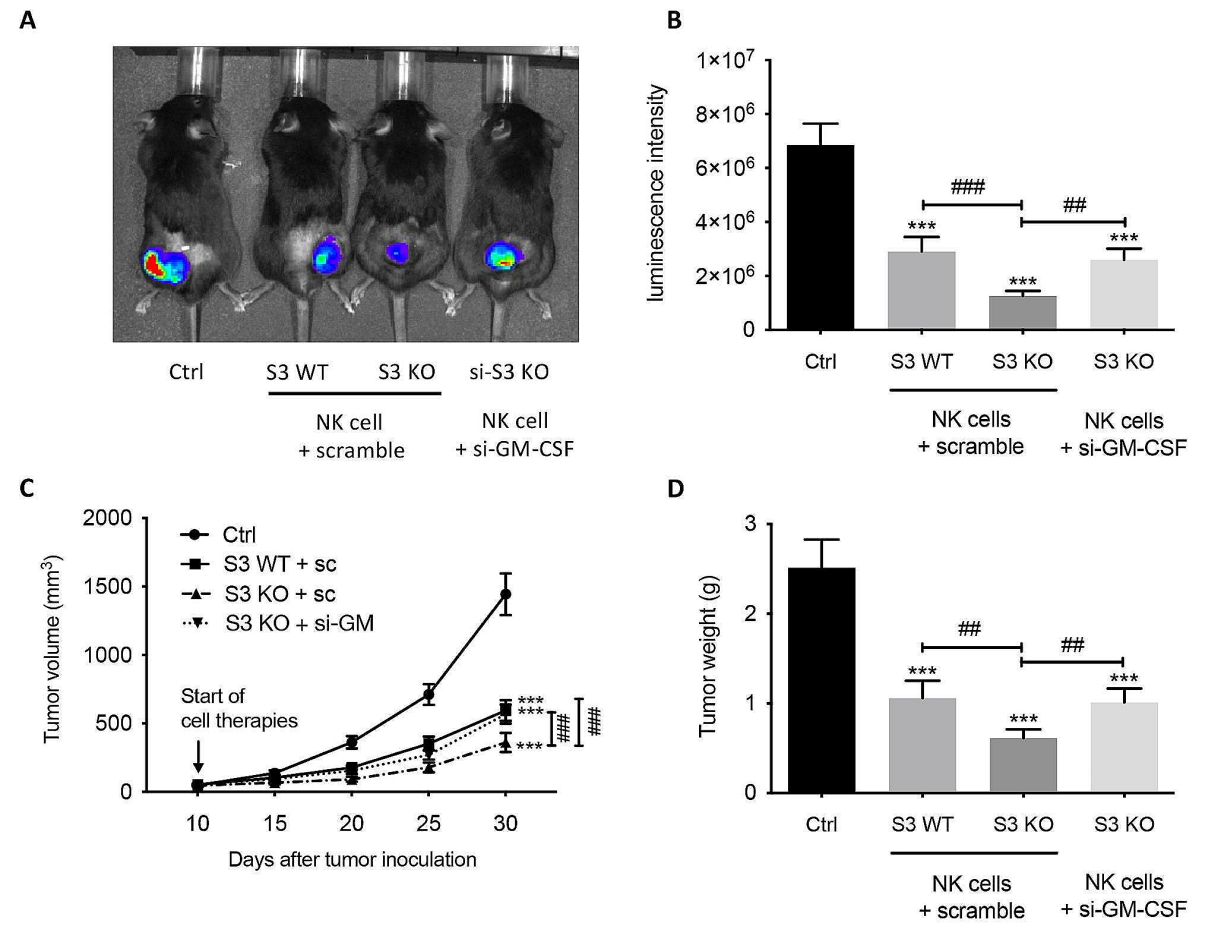
Silencing GM-CSF diminishes the therapy effects of Smad3 knockout NK cells in mouse syngeneic LLC model. **(A)** Bioluminescence imaging and **(B)** corresponding quantitative analysis of luminescent intensity. **(C)** Tumor volume measured every 5 days. **(D)** Tumor weight at day 30 since the initiation of NK cell therapies. Each bar represents the mean ± SD for groups of eight mice. *** *p* < 0.001 compared to Ctrl; ## *p* < 0.01, ### *p* < 0.001 as indicated. S3 WT: Smad3 wild-type; S3 KO: Smad3 knockout; sc or scramble: scramble sequence; si-GM-CSF: siRNA targeting GM-CSF.

Given the dichotomic functions of GM-CSF in cancer progression, we then examined the role of NK-derived GM-CSF in the progression of syngeneic Lewis Lung Cancer (LLC) in C57BL/6 mice. LLC-bearing mice were treated with either BM-NK from Smad3 wild-type mice transfected with scramble sequence, BM-NK from Smad3 knockout mice transfected with scramble sequence or BM-NK from Smad3 knockout mice transfected with short interfering RNA (siRNA) targeting GM-CSF (si-GM-CSF) once a week. Neither deletion of Smad3 nor knockdown of GM-CSF significantly influenced NK cell proliferation or apoptosis, as determined by Annexin V-PI staining and MTT assay (Suppl. Figure 1 A-C). Consistent with our previous findings [16], tumor metabolic activity in mice receiving Smad3 knockout NK cell therapy was 80% lower than mice receiving Smad3 wild-type NK cell therapy as determined by bioluminescence imaging, suggesting Smad3 significantly suppresses the anti-cancer activity of NK cells. While the bioluminescence intensity in LLC-bearing mice receiving NK cell therapy was substantially promoted when GM-CSF was silenced in Smad3 knockout NK cells, rebounding to 72% of the intensity in mice receiving Smad3 wild-type NK cell therapy (Fig. 2A and B). Correspondingly, treatment with BM-NK from Smad3 knockout mice resulted in a 2.5-fold increase in the inhibition of LLC progression, which was voided by disrupting GM-CSF production as indicated by both tumor volume and tumor weight in Fig. 2C and D.

**Fig. 3:**
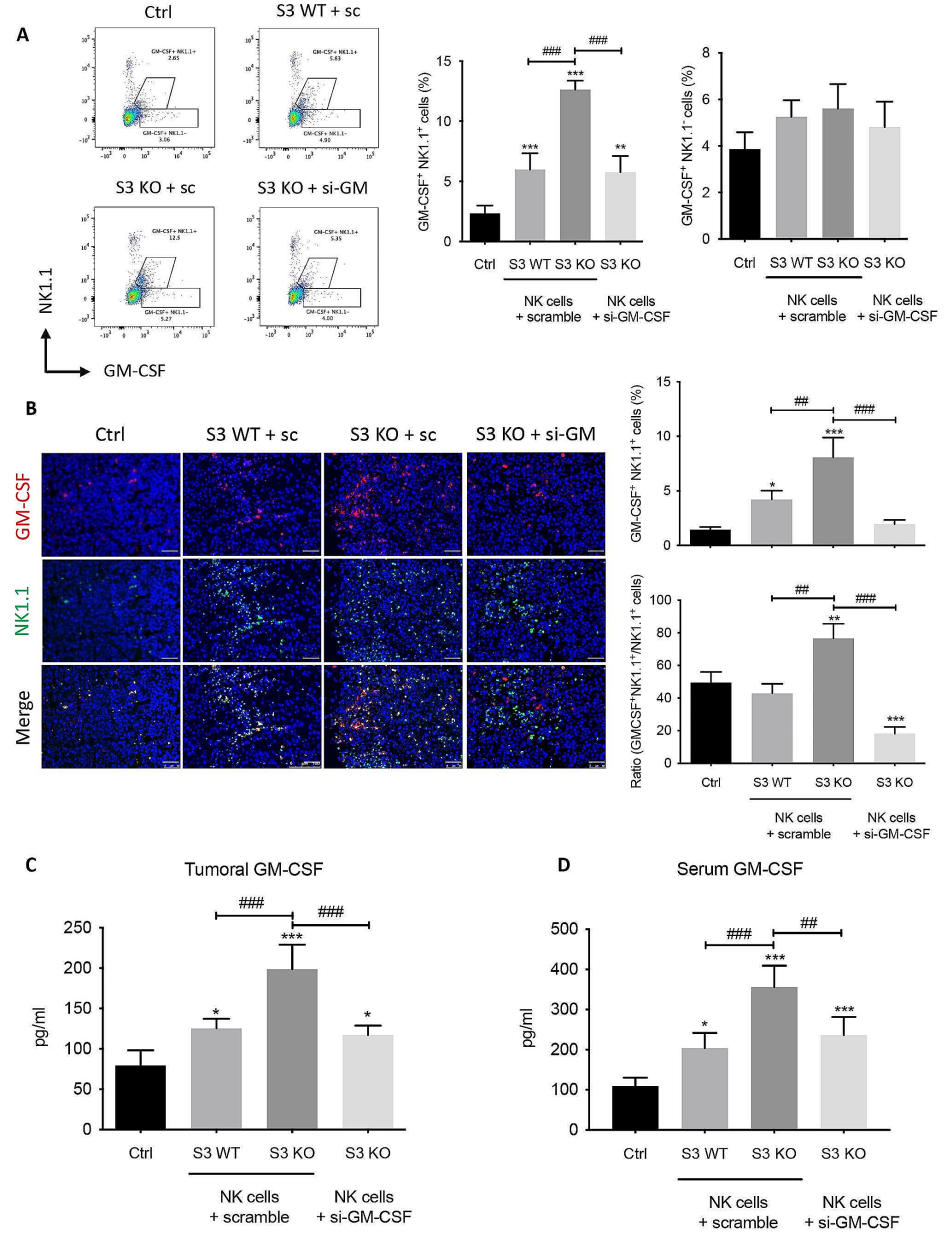
The level of GM-CSF is substantially decreased in mice receiving GM-CSF-silenced Smad3 knockout NK cell therapy. **(A)** Flow cytometry detecting GM-CSF-producing NK cells in the circulation of LLC-bearing mice receiving NK cell therapies, and corresponding quantifications. **(B)** Immunofluorescence staining detecting GM-CSF-producing NK in LLC tumor microenvironment and corresponding quantification. Scale bar, 50 μm. The levels of GM-CSF in **(C)** tumor tissue and **(D)** serum in LLC-bearing mice receiving NK cell therapies determined by ELISA. Each bar represents the mean ± SD for groups of five to six mice. * *p* < 0.05, ** *p* < 0.01, *** *p* < 0.001 compared to Ctrl; ## *p* < 0.01, ### *p* < 0.001 as indicated. S3 WT: Smad3 wild-type; S3 KO: Smad3 knockout; sc or scramble: scramble sequence; si-GM-CSF: siRNA targeting GM-CSF.

As shown in Fig. 3A, the production of GM-CSF by circulating NK cells was doubled in mice receiving Smad3 knockout-NK cell therapy compared with Smad3 wild-type NK cell therapy, however it was reduced in those receiving Smad3 knockout NK cell transfected with GM-CSF targeting siRNA. Whereas the proportions of GMCSF^+^ NK1.1^−^ cells were not significantly changed in mice with or without NK cell therapies. In parallel, comparing with mice treated with Smad3 wild-type NK cells, the proportion of GM-CSF producing NK in the tumor microenvironment was largely promoted in those treated with Smad3 knockout NK cells, but reversed in mice treated with GM-CSF-silenced Smad3 knockout NK (Fig. 3B). It should be pointed out that NK cells detected by NK1.1 staining may include a tiny amount of NKT cells. However, as shown in Suppl. Figure 2A, the proportions of NKT cells within the BM-NK cells used for cell therapy were less than 2% across all three groups. Besides, very few NKT cells were found in the tumor microenvironment of mice receiving NK cell therapies (Suppl. Figure 2B).

Meanwhile, both tumoral and serum levels of GM-CSF in mice receiving Smad3 knockout NK therapy were much higher than that in mice receiving Smad3 wild-type NK therapy, whereas they were considerably reduced in mice receiving GM-CSF-silenced Smad3 knockout NK therapy (Fig. 3C and D), illustrating NK cell as an important source for GM-CSF in LLC-bearing mice. Altogether, silencing GM-CSF in Smad3 knockout NK cells remarkably mitigated the therapeutic effects of Smad3 knockout NK cell therapy, highlighting the significance of GM-CSF in NK-mediated anti-cancer effects.

### NK-derived GM-CSF promotes DC- and M1-mediated recruitment and activation of effector T cells

**Fig. 4:**
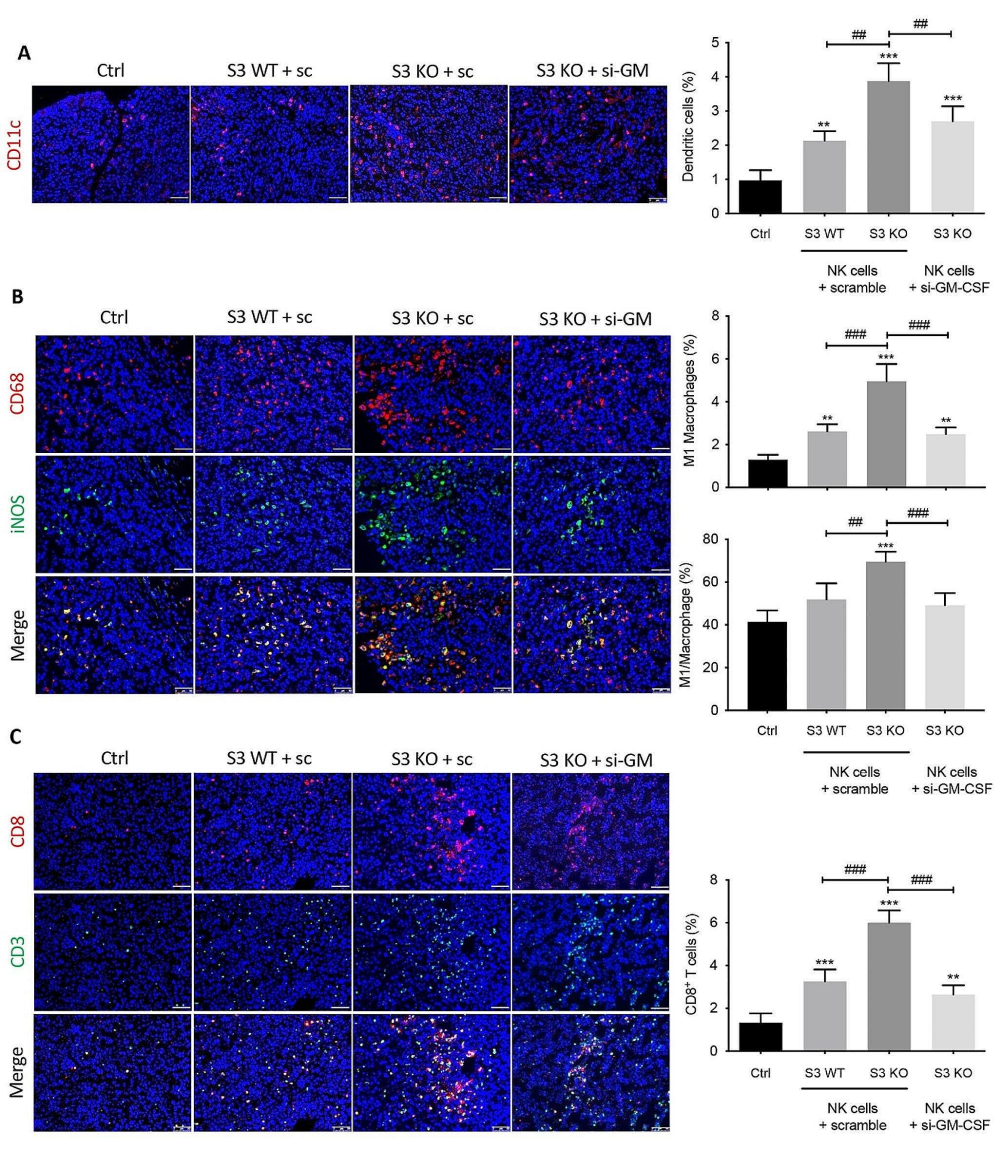
NK-derived GM-CSF encourages DC- and M1 macrophage-mediated CD8^+^ T cell activation and recruitment in lung carcinoma. Immunofluorescence staining detecting **(A)** the accumulation of DC in LLC tumor; **(B)** the accumulation of M1 macrophages in LLC tumor; and **(C)** the accumulation of CD8^+^ effector T cells in LLC tumor. Scale bar, 50 μm. Each bar represents the mean ± SD for groups of five to six mice. ** *p* < 0.01, *** *p* < 0.001 compared to Ctrl; ## *p* < 0.01, ### *p* < 0.001 as indicated. S3 WT: Smad3 wild-type; S3 KO: Smad3 knockout; sc or scramble: scramble sequence; si-GM-CSF: siRNA targeting GM-CSF.

As GM-CSFR is mostly expressed on myeloid cells such as DC, monocytes and macrophages, the anti-cancer GM-CSF effects are largely attributed to the influx and activation of DC and pro-inflammatory M1 macrophages, which subsequently activate T cells through antigen presenting in the tumor tissue [34]. Therefore, we evaluated the impact of silencing GM-CSF in Smad3 knockout NK cells as a cell therapy on the recruitment of DC, M1 macrophages and T cells in LLC microenvironment. In comparison with Smad3 wild-type NK cell therapy, the accumulation of DC in LLC tumor was increased twofold by Smad3 knockout NK cell therapy (Fig. 4A). Nevertheless, this elevation was abolished by the knockdown of GM-CSF in Smad3 knockout NK cells. Similarly, the number of tumor-infiltrated iNOS^+^ M1 was markedly increased in mice receiving Smad3 knockout NK cell therapy as compared with those receiving Smad3 wild-type NK cell therapy. Likewise, reducing NK-derived GM-CSF with si-RNA also nullified the protective effects of Smad3 knockout NK cell therapy on the recruitment of M1 macrophages, which was aligned with the changes of GM-CSF levels in the tumor tissue (Fig. 3C).

Compared with mice treated with Smad3 wild-type NK cells, the recruitment of CD8^+^ T cells was largely enhanced in mice treated with Smad3 knockout NK cells. Moreover, the proportion of effector CD8^+^ T cells was additionally influenced by NK-derived GM-CSF, as silencing GM-CSF in Smad3 knockout NK cells notably abrogated the recruitment of CD8^+^ T cells (Fig. 4C). Overall, Smad3-mediated inhibition of GM-CSF production in NK cells significantly impeded the recruitment of DCs and M1 macrophages in LLC tumor, which consequently reduced effector T cells in LLC microenvironment.

As high dose GM-CSF may play a pathogenic role by stimulating the differentiation of immunosuppressive MDSC and Treg cells [35], we further evaluated the influence of NK cell therapies on the accumulation of MDSCs and Tregs in the LLC tumor tissue. As shown in Suppl. Figure 3A, the amount of tumor-infiltrated MDSC was enhanced by Smad3 knockout NK cell therapy from 17 to 23%, but not by Smad3 wild-type NK cell therapy nor GM-CSF-silenced Smad3 knockout NK cell therapy. Meanwhile, the percentage of Treg cells was increased from 4.7 to 5.7% compared with control by Smad3 wild-type NK cell therapy, and to 7.2% by Smad3 knockout NK therapy, while blocking GM-CSF production by these NK cells largely mitigated the pathogenic effect, resulting in a reduction in Treg proportion to 5.5% of total tumor-infiltrated leukocytes (Suppl. Figure 3B).

### NK-derived GM-CSF promotes the survival of tumor-infiltrated neutrophils, which subsequently triggers the terminal maturation and promotes the anti-cancer functions of NK cells in positive feedback circuit

**Fig. 5:**
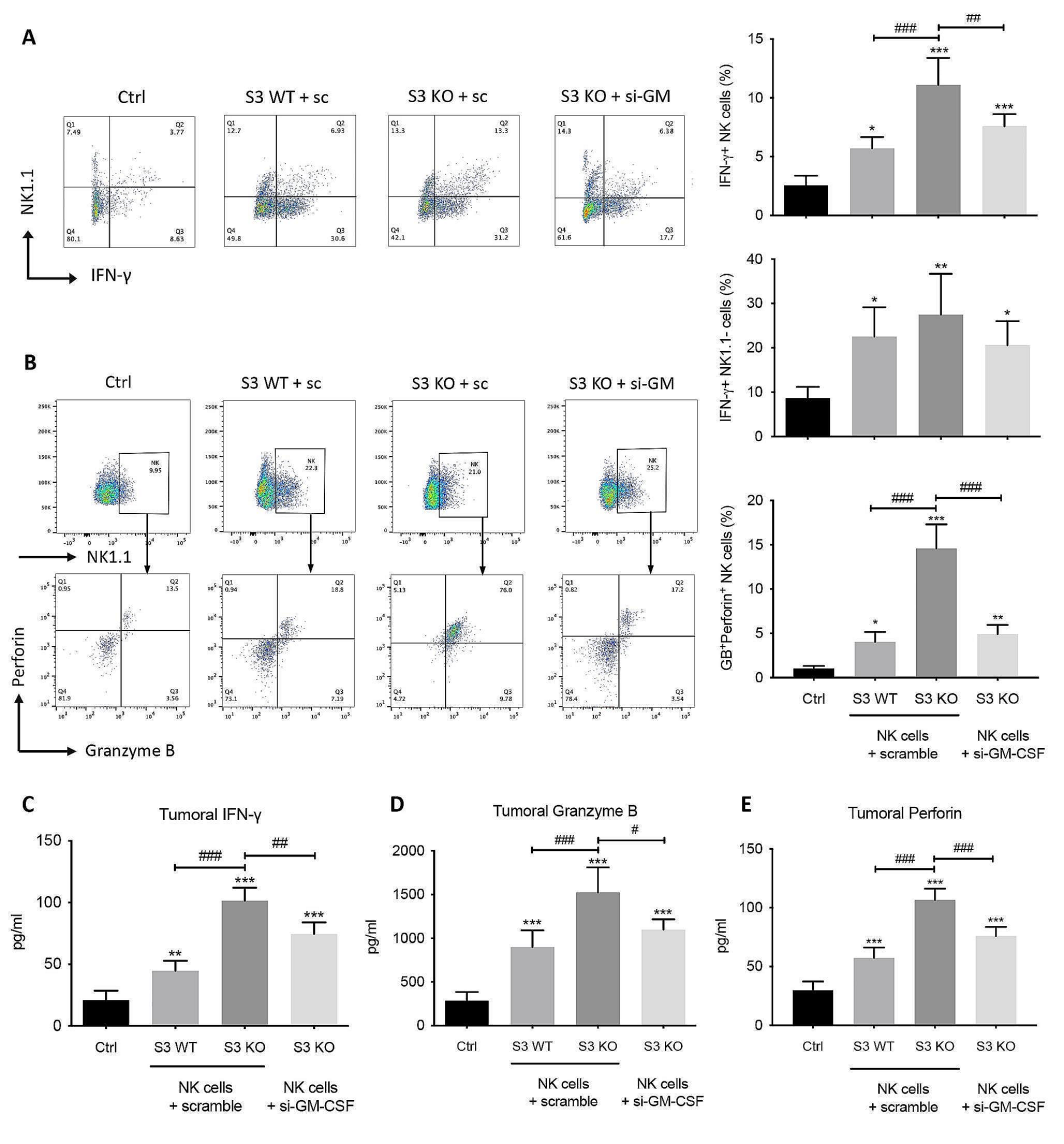
GM-CSF augments the cytotoxic functions of NK cells in LLC-bearing mice. **(A)** Flow cytometry examining the proportion of IFN-γ producing NK in the circulation. **(B)** Flow cytometry detecting the proportion of Granzyme B and perforin producing cytolytic NK in the circulation **(C-E)** The levels of (C) IFN-γ, (D) granzyme B and (E) perforin in LLC tumor were analyzed by ELISA. Each bar represents the mean ± SD for groups of five to six mice. * *p* < 0.05, ** *p* < 0.01, *** *p* < 0.001 compared to Ctrl; # *p* < 0.05, ## *p* < 0.01, ### *p* < 0.001 as indicated. S3 WT: Smad3 wild-type; S3 KO: Smad3 knockout; sc or scramble: scramble sequence; si-GM-CSF: siRNA targeting GM-CSF.

In addition to T cells, NK cells also serve as potent tumor killers through direct killing and cytokine-mediated cellular crosstalk. To explore whether NK-derived GM-CSF also regulates NK-mediated immune responses in the tumor microenvironment, we analyzed the effector functions of NK cells in LLC-bearing mice after 3-week NK cell therapies. As shown in Fig. 5A, the proportion of IFN-γ producing NK in the circulation of mice treated with Smad3 knockout NK cells was doubled compared to those treated with Smad3 wild-type NK cells, yet the proportion was decreased to half when GM-CSF was knocked down in the Smad3 knockout NK cells. We also observed the proportion of IFN-γ^+^ NK1.1^−^ cells was remarkably enhanced by Smad3 knockout NK cell therapy compared with wild-type NK therapy, but it was largely reduced by GM-CSF knockdown. As the numbers of NKT cells (Fig. Suppl. Figure 2), CD8^−^ T cells (Fig. 4) and Treg cells (Fig. Suppl. Figure 3B) were not remarkably changed by NK cell therapies, it is possible that the majority of the IFN-γ^+^ NK1.1^−^ cells in Fig. 5A are cytotoxic T cells. To confirm this, IFN-γ and CD8 were co-stained in the tumor microenvironment. As shown in Suppl. Figure 4, the amount of IFN-γ-producing CD8^+^ T cells in the tumor tissue remarkably increased in mice receiving Smad3 knockout NK cell therapy compared with that receiving Smad3 wild-type NK therapy, while the increase was reduced by knocking down GM-CSF in Smad3 knockout NK cells.

Comparably, the percentage of cytolytic NK cells among total NK cells, characterized by the productions of granzyme B and perforin, in mice receiving GM-CSF-silenced Smad3 knockout NK cells, was also notably reduced to 33% of those receiving scramble sequence-transfected Smad3 knockout NK cells (Fig. 5B). Since the amount of effector T cells and cytolytic NK cells, being the two major sources of IFN-γ, granzyme B and perforin, were correlated with the production of GM-CSF by NK cells used for adoptive transfer therapies, we hypothesized that the levels of these cytokines would also be associated with the level of NK-derived GM-CSF in the tumor microenvironment. As shown in Fig. 5C-E, the enhancive effects on the production of IFN-γ, granzyme B and perforin induced by disrupting Smad3 in NK cells were largely impaired by knocking down GM-CSF, implying that NK-derived GM-CSF was crucial for NK- and cytotoxic T cell-mediated cytotoxicity in LLC microenvironment.

To determine whether GM-CSF produced by NK cells can in turn directly regulate the cytotoxic functions of NK cells, the productions of IFN-γ, granzyme B and perforin in GM-CSF-silenced BM-NK cells were assessed by ELISA. According to the results in Suppl. Figure 5A-C, the levels of IFN-γ, granzyme B and perforin in the supernatant of both GM-CSF-silenced (si-GM) Smad3 wild-type and Smad3 knockout BM-NK cells were comparable to those in BM-NK cells transfected with scramble sequence. Moreover, stimulation with mouse recombinant GM-CSF also did not affect the production of these cytotoxic effectors by either Smad3 wild-type or Smad3 knockout BM-NK cells (Suppl. Figure 5D-F). These results suggested that GM-CSF could not regulate the effector functions of NK cells directly.

**Fig. 6:**
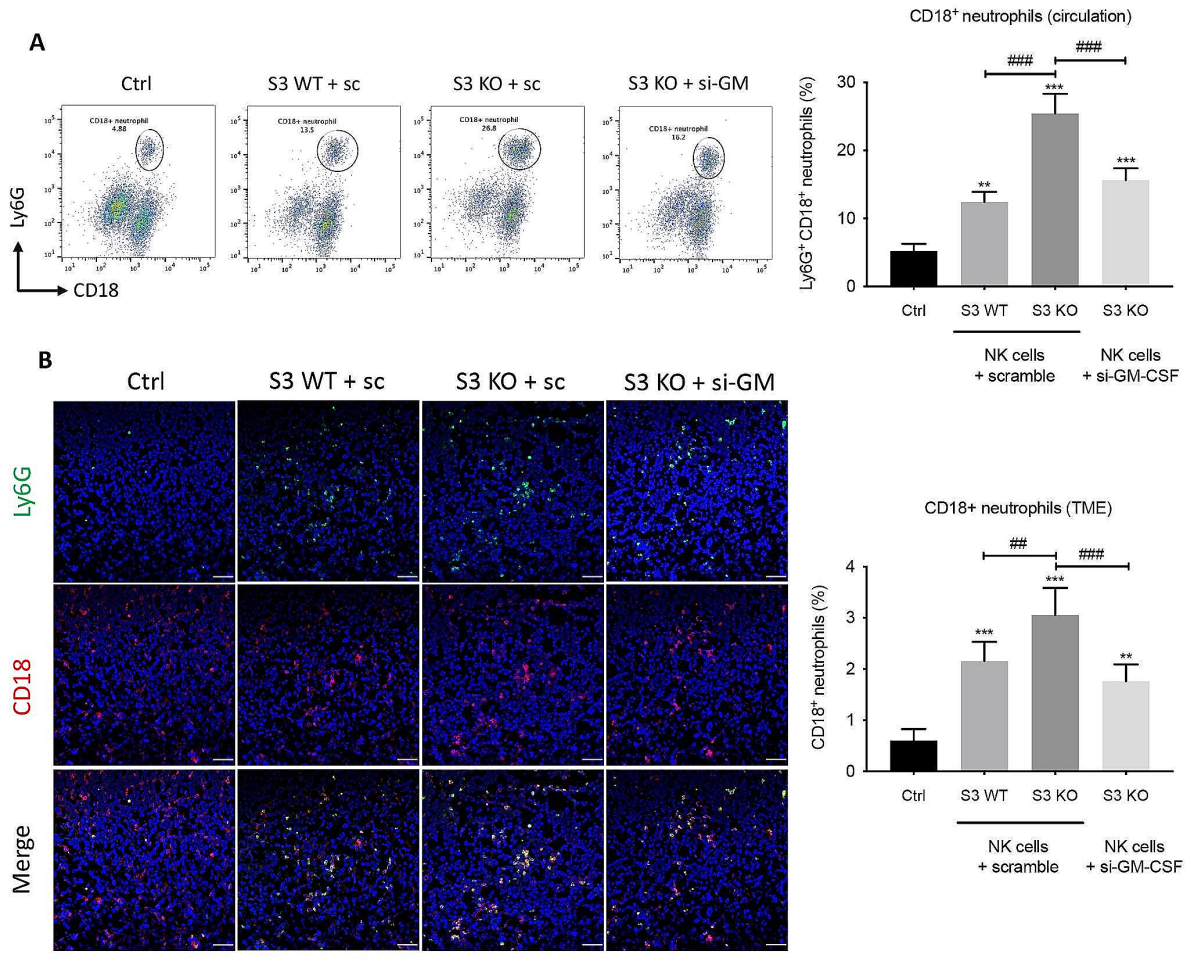
NK-derived GM-CSF is essential for CD18^+^ neutrophil survival in LLC-bearing mice. **(A)** The proportion of CD18^+^ Ly6G^+^ neutrophils in the circulation analyzed by flow cytometry. **(B)** The proportion of tumor-infiltrated CD18^+^ Ly6G^+^ neutrophils in LLC-bearing mice analyzed by immunofluorescence. Each bar represents the mean ± SD for groups of five to six mice. ** *p* < 0.01, *** *p* < 0.001 compared to Ctrl; ## *p* < 0.01, ### *p* < 0.001 as indicated. S3 WT: Smad3 wild-type; S3 KO: Smad3 knockout; sc or scramble: scramble sequence; si-GM-CSF: siRNA targeting GM-CSF.

Jaeger et al. revealed that neutrophils play a critical role in the terminal maturation of NK cells, characterized by the expression of CD11b and CD43, and their corresponding effectiveness against cancer [36]. Through the interaction of CD18/ICAM-3, neutrophils directly bind to NK cells, accelerating their terminal maturation and promoting IFN-γ production [37]. Meanwhile, GM-CSF promotes the survival of neutrophil by suppressing their apoptosis [38]. These lead us to the hypothesis that NK-derived GM-CSF prolongs the lifespan of neutrophils, which consequently facilitates the maturation and boosts the cytotoxicity of NK cell to form a positive anti-cancer feedback loop. To validate this hypothesis, we determined the amount of CD18^+^ neutrophils in the circulation and tumor tissue after NK cell therapies. Compared with mice without treatment, cell therapy with Smad3 wild-type NK cells increased the number of neutrophils in circulation from 5 to 12%, and the amount was further doubled by treatment with Smad3 knockout NK cells (Fig. 6A). However, silencing GM-CSF in Smad3 knockout NK cells significantly blunted the protective effect of these NK cells, which lowered the proportion of CD18^+^ neutrophils from 25% back to 15.6%, indicating NK-derived GM-CSF was important for CD18^+^ neutrophil survival in the circulation. Similar effects were also observed in the LLC tumor, where the accumulation of CD18^+^ neutrophils was significantly enhanced by Smad3 knockout NK therapy compared with Smad3 wild-type NK therapy. While the number was substantially dropped when GM-CSF was silenced in Smad3 knockout NK therapy, which was even lower than that in mice treated with Smad3 wild-type NK therapy (Fig. 6B). Thereby, our data suggested NK cell prolonged the survival of CD18^+^ neutrophils in LLC-bearing mice by producing GM-CSF.

**Fig. 7:**
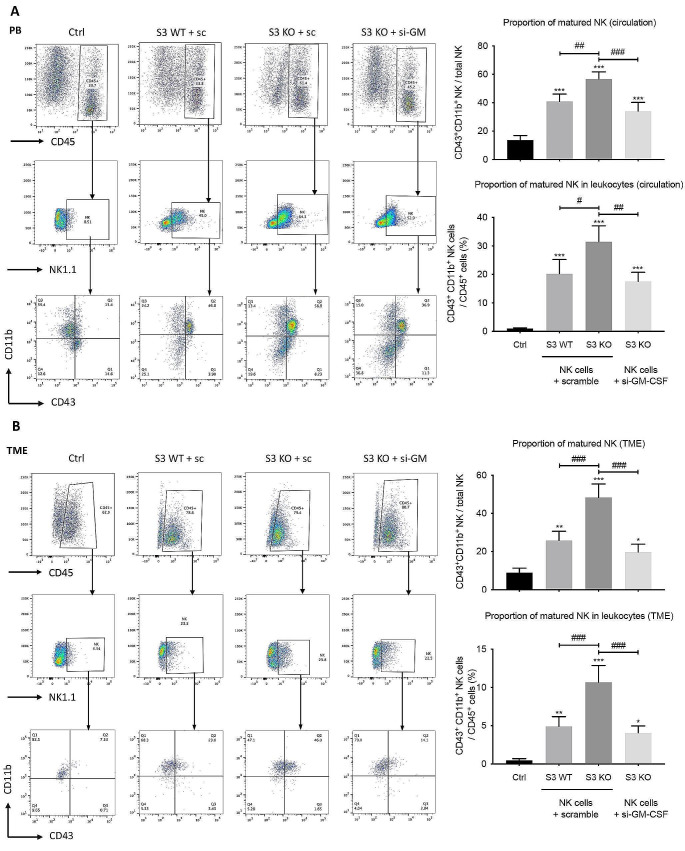
Silencing GM-CSF in Smad3 knockout NK cells used for cell therapy impedes the maturation of NK cells in LLC-bearing mice. **(A)** The proportion of CD11b^+^ CD43^+^ matured NK in the circulation and corresponding quantifications analyzed by flow cytometry. **(B)** The proportion of CD11b^+^ CD43^+^ matured NK in LLC tumor and corresponding quantifications analyzed by flow cytometry. Each bar represents the mean ± SD for groups of five to six mice. * *p* < 0.05, ** *p* < 0.01, *** *p* < 0.001 compared to Ctrl; # *p* < 0.05, ## *p* < 0.01, ### *p* < 0.001 as indicated. S3 WT: Smad3 wild-type; S3 KO: Smad3 knockout; sc or scramble: scramble sequence; si-GM-CSF: siRNA targeting GM-CSF.

Next, we examined whether the terminal maturation of NK cells was affected by the level of NK-derived GM-CSF in the LLC mice. As shown in Fig. 7A, the proportion of CD11b^+^ CD43^+^ matured NK cells in total NK cells in the circulation of LLC-bearing mice was raised from 9 to 26% by Smad3 wild-type NK cell therapy, and it was further elevated to 48% by Smad3 knockout NK cell therapy. However, silencing GM-CSF in Smad3 knockout NK cells remarkably reduced the proportion of matured NK cells to only 20%, indicating that NK-derived GM-CSF was critical for the terminal maturation of circulating NK cells in LLC-bearing mice. Although the number of tumor-infiltrated NK 1.1^+^ cells was comparable in mice treated with either NK cell therapy, the percentages of matured CD11b^+^ CD43^+^ NK in both tumor-infiltrated NK cells and tumor-infiltrated CD45^+^ leukocytes were increased twofold by Smad3 knockout NK cell therapy compared with Smad3 wild-type NK cell therapy, while the increments were also abolished when GM-CSF was knocked down in Smad3 knockout NK cells (Fig. 7B). Such findings supported our hypothesis that NK-derived GM-CSF was essential for neutrophil survival, which subsequently encouraged the terminal maturation of NK cells and enhanced their effector functions against cancer.

## Discussion

The disruption of Smad3 in NK cells significantly strengthens the therapeutic effects of NK cell therapy [14, 16]. Apart from the powerful killing capacity, NK cells also play an essential role in shaping the immune microenvironment via cellular crosstalk with cytokines and chemokines [39]. Nevertheless, the role of Smad3 on the immunoregulatory functions of NK cells is yet to be explored. In the present study, we found the disruption of Smad3 substantially enhanced the production of GM-CSF by NK cells without influencing their proliferation nor apoptosis. Promoter analysis identified Smad3 as a transcriptional repressor for *CSF2* gene, which was validated by ChIP assay and luciferase reporter assay. Functionally, we noticed NK-derived GM-CSF effectively boosted T cell-mediated immune response against cancer through the recruitment of DC and pro-inflammatory macrophage. Moreover, GM-CSF prolonged the survival of neutrophils, which sequentially interacted with NK cells to facilitate the terminal maturation and potentiate their cancer killing effects. Thereby, silencing GM-CSF in Smad3 knockout NK cells considerably blunted its immunomodulatory effects by suppressing DC- and macrophage-mediated activation of T cells, as well as impairing the positive feedback loop between neutrophils and NK cells. Taken together, on top of directly promoting NK cell-mediated cytotoxicity against cancer cells, blocking Smad3 augmented the immunostomulatory effects of NK cells by enhancing the production of GM-CSF.

Despite being a potent immunomodulator for DC maturation and macrophage activation, GM-CSF is also notorious for its pro-tumoral functions by stimulating the development of MDSC and recruiting of Treg cells in tumor microenvironment. Recent findings suggested that the role of GM-CSF on tumor progression greatly depends on the dosage, immune microenvironment and cancer types [40]. Higher dose of GM-CSF in the system but not in the tumor site is associated with the accumulation of MDSC and impaired T cell immune responses [41]. Therefore, a sustained local release strategy such as GM-CSF-secreting tumor vaccine or through engineering of GM-CSF-producing immune cells may help to reduce the immunosuppressive effects while exploiting the immunostimulatory functions of GM-CSF. In the present study, blocking of Smad3 signaling notably increased the production of GM-CSF by tumor-infiltrated NK cells, serving as a long-lasting source of GM-CSF in the tumor microenvironment. However, it should be noted that Smad3 knockout NK cell therapy did slightly enhance the accumulation of MDSC and Treg cells in LLC tumor, which indicated NK-derived GM-CSF may induce moderate immunosuppression in lung carcinoma. But this immunosuppressive effect was countered by the potent immunostimulatory functions of NK-derived GM-CSF, as silencing GM-CSF markedly impaired the anti-cancer effects of Smad3 knockout NK cells.

While being recognized as a M1 inducer, GM-CSF may also trigger the polarization of macrophage towards M2 phenotype by inducing the expression of CCR2 [42]. Yet our data showed that blocking GM-CSF in Smad3 knockout NK cells significantly reduced the proportion of iNOS-producing M1 in tumor-infiltrated macrophages, illustrating that NK-derived GM-CSF exerts pro-inflammatory functions in the LLC microenvironment.

Not until recent decades, the immunoregulatory role of neutrophils has been neglected due to their short lifespan [43]. In this study, we observed the changes in the proportion of neutrophils in the tumor microenvironment by blocking Smad3 and GM-CSF in NK cells were comparable to those in the circulation. This indicates that the effect of NK-derived GM-CSF on the accumulation of CD18^+^ neutrophils may not be associated with their infiltration but survival. This result is consistent with previous studies that found NK cells effectively prolong the survival of neutrophils by inducing resistance to apoptosis with GM-CSF [35]. Mechanistically, GM-CSF protects neutrophils from apoptosis by inducing the expression of Bcl-2 family members Mcl-1 and A1/Bfl-1, through the activation of p42/p44 MAPK, PI3K and JAK/STAT signalings [44, 45].

However, it needs to be noted that neutrophils have multifaceted roles and high plasticity in the tumor microenvironment. Apart from its anti-tumoral activities, neutrophils also induces immunosuppression in a TGF-β-dependent manner, as well as promoting tumor proliferation and metastasis through releasing neutrophil extracellular traps (NETs) [46]. Nevertheless, the pro-inflammatory microenvironment with high levels of IFN-γ and GM-CSF could drive the polarization of neutrophil towards an APC-like phenotype with enhanced anti-tumor activities, characterized by the expression of co-stimulatory molecules OX40L, CD86 and 4-1BBL, which also possesses antigen presenting capability to stimulate T cell-mediated immune responses in the early stage of lung carcinoma [47]. Thus, by increasing the production of IFN-γ and GM-CSF, Smad3-silenced NK cell therapy may promote the polarization of APC-like neutrophils with enhanced anti-cancer effects and immunostimulatory functions. The activation of anti-tumor T cell response may result in further enhanced production of IFN-γ in the tumor microenvironment, which reinforces APC-like neutrophils polarization in a positive feedback pathway.

Interestingly, we found that NK1.1 expression level in GM-CSF^+^ NK cell was lower compared with GM-CSF^−^ NK cell. This indicates that NK1.1^dim^ NK cells may function as the immunoregulatory subtype compared with their NK1.1^bright^ counterpart. But this speculation is yet to be validated. Besides, we also noticed the participation of TGF-β-dependent Smad-independent regulations on the production of GM-CSF, as TGF-β stimulation was also found to suppress the secretion of GM-CSF in NK-92-S3KD, BM-NK cells and splenic NK cells isolated from Smad3 knockout mice. Previous studies discovered that PI3K and MAPK/ERK pathways also involve in the transcriptional or post-transcriptional regulations of GM-CSF [48, 49], suggesting TGF-β may regulate the expression of GM-CSF through crosstalk with PI3K and MAPK/ERK pathways in Smad-independent manners.

Ever since Dranoff et al. engineered GM-CSF-secreting melanoma cell as a potential long-lasting and safe therapy in 1993, GM-CSF-producing oncolytic tumor vaccine has been regarded as a promising therapeutic strategy for various types of cancer [50]. Despite the failure to generate satisfying clinical outcomes as monotherapy [51], GM-CSF-secreting tumor vaccines function in a synergistic way with immune checkpoint inhibitors (ICIs) such as Ipilimumab (anti-CTLA-4 antibody) in metastatic melanoma by virtue of its mighty immunostimulatory effects [52]. Therefore, NK-92-S3KD cell also represents as a promising cell therapy in combination with ICIs or other immunotherapies given its robust cytotoxicity against cancer cells and powerful immunomodulatory capacity in the TGF-β-rich microenvironment.

## Methods

### NK cell culture

NK-92 cells (CRL-2407, ATCC) were cultured in MEM-α medium (Gibco, Thermo Fisher Scientific) supplemented with 12.5% fetal bovine serum (FBS), 12.5% horse serum (Gibco, Thermo Fisher Scientific), 50 mM β-mercaptoethanol (Gibco, Thermo Fisher Scientific), 0.2mmol/L inositol, 0.02mmol/L folic acid (Sigma-Aldrich), and 20 ng/ml IL-2 (PeproTech).

To induce bone marrow-derived NK cells, femurs from Smad3 wild-type or Smad3 knockout mice were collected for bone marrow cell isolation. The cells were cultured in MEM-α medium containing 10% FPS, supplemented with 1 ng/mL IL-7, 10 ng/mL Flt3 ligand, 30 ng/mL stem cell factor, 50 ng/mL IL-15 (PeproTech) and 50 mM β-mercaptoethanol (Gibco, Thermo Fisher Scientific) for 4 days. Then the immature NK cells were transferred to MEM-α medium containing 10% FBS supplemented with 50 ng/mL IL-15, 20 ng/mL IL-2, and 50 mM β-mercaptoethanol to induce the maturation for another week.

### Generating stable Smad3-knockdown cell line (NK-92-S3KD) with lentiviral transfection

To prepare NK-92-EV or NK-92-S3KD cells, either a pLVX-shRNA1-Puro plasmid as empty vector or a recombinant plasmid containing shRNA specifically targeting Smad3 was transfected into 293T cells to produce recombinant lentivirus particles. The recombinant lentivirus was then transduced into NK-92 cells at MOIs equal to 50. To enhance the transduction efficiency, cells were centrifuged at 1800 rpm for 45 min. Then the cells were selected with 2 µg/ml puromycin (InvivoGen). The polyclonal resistant cells were expended and validated with real-time PCR and Western blot. The inhibitory efficiency on the expression of Smad3 is over 70% as described previously [16].

### Knockdown GM-CSF in BM-NK cells with siRNA

To silence GM-CSF in BM-NK cells, scramble sequence or si-RNA specifically targeting GM-CSF (sense 5’- GCCAGCUACUACCAGACAUTT-3’; anti-sense 5’- AUGUCUGGUAGUAGCUGGCTT-3’) was transfected into BM-NK cells with lipofectamine RNAiMAX (Invitrogen) for 24 h before the cells were used for adoptive cell therapies.

### Cytokine assay and ELISA

The supernatant of NK-92-EV or NK-92-S3KD cells cultured in 6-well plate for 24 h was collected respectively for cytokine assay using Human Cytokine Array C5 (RayBiotech).

To examine the regulation of TGF-β on the production of GM-CSF, NK cells, NK-92 cells, BM-NK cells and splenic NK cells were stimulated with 5 ng/ml recombinant TGF-β1 (R&D Systems) for 24 h, and supernatant was collected respectively. Tumor homogenates were prepared by homogenizing tumor tissue with chilled PBS supplemented with protease inhibitor cocktail (Thermo Fisher Scientific). The levels of GM-CSF, IFN-γ (R&D system), granzyme B and perforin (eBioscience, Thermo Fisher Scientific) were then measured by ELISA.

### Chromatin immunoprecipitations (ChIP) assay

NK-92 cells were stimulated with 5 ng/mL recombinant TGF-β1 for 1 hour, followed by cross-linking with 37% formaldehyde and isolation of total chromatin with SimpleChIP Enzymatic Chromatin IP Kit (Cell Signaling Technology, MA, USA). The cross-linked DNA fragments were precipitated with anti-phospho-Smad3 antibody (Cell Signaling Technology, MA, USA) or anti-normal rabbit IgG antibody (Cell Signaling Technology). After DNA purification, the cross-linked DNA fragments were analyzed with PCR amplification using specific primers targeting the predicted Smad binding site: forward 5’-GGATGGGGAGCCCTATCTAA-3’ and reverse 5’-AACCTTTCCAAGAACCGACA-3’.

### Luciferase reporter assay

The human Smad3 coding sequence was cloned into a pcDNA3.1 plasmid as pcDNA3.1 + wt-Smad3. Then a point mutation was induced in the *Smad3* gene with KOD -Plus- Mutagenesis Kit (Toyobo) as pcDNA3.1 + mut-Smad3, and the mutated site was confirmed with DNA sequencing. We also introduced a point mutation on the predicted Smad binding site (5’-AGACTGCCC-3’) with DpnI (Promega, WI, USA) and confirmed the desired mutation with DNA sequencing. The original and the mutated sequences of the promoter region of *CSF2* gene were clone into a pGL3 luciferase reporter basic vector respectively as pGL3-CSF2 and as pGL3-mut-CSF2.

To perform the luciferase reporter assay, either pGL3 basic plasmid, pGL3-CSF2 plasmid or pGL3-mut-CSF2 plasmid was transfected into Hela cells with Lipofectamine 2000 (Invitrogen). Meanwhile, either pcDNA3.1 + wt-Smad3 or pcDNA3.1 + mut-Smad3 was co-transfected into the cells to overexpress human Smad3 for 24 h. Then the luciferase activity was evaluated with Dual-Luciferase Reporter Assay System (Promega, WI, USA).

### Lewis lung cancer mouse model and NK cell therapy

LLC cells (CRL-1642, ATCC) labelled with luciferase were cultured in DMEM (Gibco, Thermo Fisher Scientific) supplemented with 10% FBS. To establish the syngeneic Lewis Lung Cancer model, 2 × 10^6^ LLC cells were inoculated onto the flank of 8-week male C57BL/6 mice purchased from The Chinese University of Hong Kong Laboratory Animal Services Centre. When tumor size reached 50 mm^3^, the mice were randomly divided into 4 groups, namely control group, Smad3 wild-type-BM-NK treatment group, Smad3 knockout-BM-NK treatment group and GM-CSF silenced Smad3 knockout-BM-NK treatment group. The mice were treated with 5 × 10^6^ BM-NK cells from Smad3 wild-type or Smad3 knockout mice transfected with scramble sequence as mentioned above, or BM-NK cells from Smad3 knockout mice transfected with GM-CSF-specific si-RNA intravenously once a week from day 10 after tumor inoculation, followed by intraperitoneal injection of 10 ng/g IL-2 to maintain the survival of NK cells. All the animal experiments were performed following a protocol approved by Animal Ethics Experimental Committee at the Chinese University of Hong Kong.

Tumor volume was measured every 5 days according to formula: volume = width × length × height × 1/2. Tumor activity was measured 15 min after intraperitoneal injection of D-Luciferin (Sigma-Aldrich) with bioluminescence imaging using the IVIS Spectrum system (Xenogen, PerkinElmer) on day 30 after tumor inoculation.

### Immunofluorescence staining

Immunofluorescence staining was performed with PLP-fixed frozen sections. After drying at room temperature for 30 min, the frozen sections were blocked with 5% BSA/PBS for 30 min and incubated with anti-GMCSF, FITC-conjugated anti-NK1.1, PE-conjugated anti-CD11c, FITC-conjugated anti-CD8, Alexa 488-conjugated anti-iNOS (eBioscience, Thermo Fisher Scientific), Alexa 594-conjugated anti-CD68, FITC-conjugated anti-Ly6G, Alexa 594-conjugated anti-CD18 (BioLegend), PE-conjugated anti-CD3 (BD Biosciences) antibodies diluted at the ratio of 1:100 in staining buffer overnight at 4 °C, followed by 2 h-incubation with Alexa 594-conjugated anti-rabbit secondary antibody (Invitrogen, Thermo Fisher Scientific) diluted at the ratio of 1:500 in staining buffer for GM-CSF staining sections. After washing with PBS for 3 times, the sections were mounted with Hoechst 33,342 (Invitrogen, Thermo Fisher Scientific) -containing SlowFade Gold Antifade Mountant (Invitrogen, Thermo Fisher Scientific) and analysed under DM6000 M fluorescence microscope (Leica Microsystems) or LSM 880 confocal microscope (Carl Zeiss).

### Flow cytometry

Peripheral blood mononuclear cells from LLC-bearing mice were isolated by Percoll density gradient centrifugation (Sigma-Aldrich). LLC tumor tissue was digested with blenzyme 4 (Roche Inc.) for 30 min at 37 °C and prepared into single cell suspension. Then the cells were fixed with IC Fixation Buffer (eBioscience, Thermo Fisher Scientific) for 30 min, and then incubated with antibodies including PE-conjugated anti-GMCSF (1:100 dilution, eBioscience), APC-conjugated anti-NK1.1 (1:100 dilution, eBioscience), PE-conjugated anti-CD4 (1:100 dilution, eBioscience), APC-conjugated anti-CD25 (1:100 dilution, eBioscience), PE-conjugated anti-IFN-γ (1:100 dilution, eBioscience), PE-conjugated anti-granzyme B (1:100 dilution, eBioscience), APC-conjugated anti-perforin (1:25 dilution, eBioscience), FITC-conjugated anti-Gr-1 (1:200 dilution, eBioscience), FITC-conjugated anti-NK1.1 (1:100 dilution, BioLegend), APC-conjugated anti-Ly6G (1:200 dilution, BioLegend), Alexa 594-conjugated anti-CD18 (1:100 dilution, BioLegend), APC-conjugated anti-CD11b (1:100 dilution, BD Biosciences), PE-conjugated anti-CD43 (1:100 dilution, BD Biosciences) antibodies and the corresponding isotype control antibodies diluted at the ratio of 1:100 in flow cytometry staining buffer (eBioscience) at 4 °C for 30 min. After washing with PBS for 3 times, cells were resuspended with 500 mL PBS and analyzed by FACSCalibur flow cytometer (BD Biosciences).

### Statistics

All data used in this study were analysed with GraphPad Prism 8.4 software by one-way ANOVA for single-variable analysis or two-way ANOVA for two in-dependent variables analysis, followed by Tukey’s or Bonferroni multiple comparisons tests.

### Electronic supplementary material

Below is the link to the electronic supplementary material.

Supplementary Material 1

## Data Availability

All data supporting the findings of this study are available in the paper or the supplementary materials.
