# Supplementary material for: Disrupting Smad3 potentiates immunostimulatory function of NK cells against lung carcinoma by promoting GM-CSF production
Source: Cell Mol Life Sci. 2024 Jun 15;81(1):262. doi: 10.1007/s00018-024-05290-4 (PMC11335298; doi:10.1007/s00018-024-05290-4)
Supplement: Supplementary file 1 — Supplementary Material 1 [file 18_2024_5290_MOESM1_ESM.pdf]

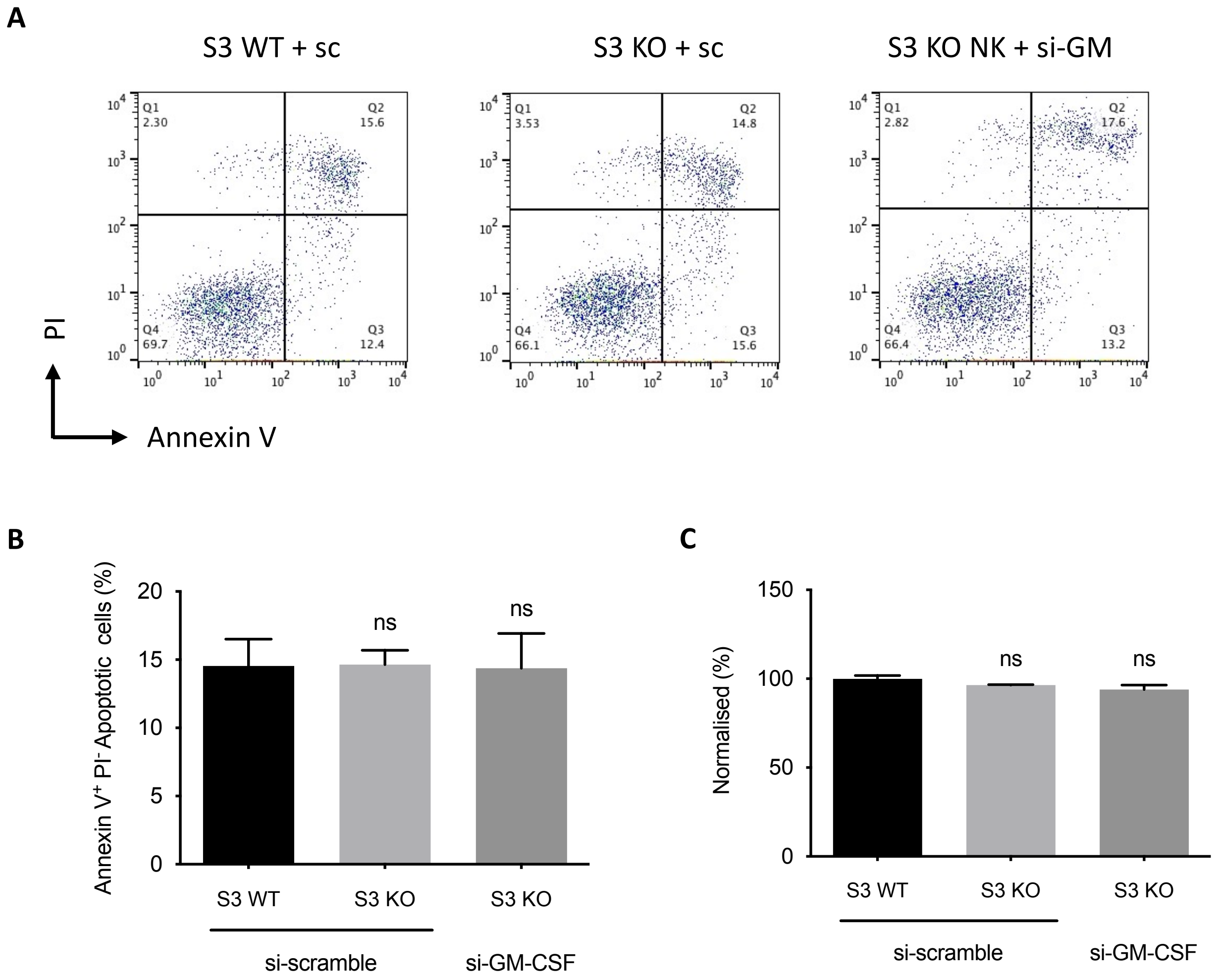

Figure S1. Neither knockout Smad3 nor knockdown GM-CSF affect cell proliferation or apoptosis in BM-NK cells . (A-B) Flow cytometry analysing apoptotic BM-NK cells with Annexin V and PI staining and quantification results. **(C)** MTT assay evaluating BM-NK cell proliferation. Each bar represents the mean ± SEM for groups of three independent experiments. ns, not significant.

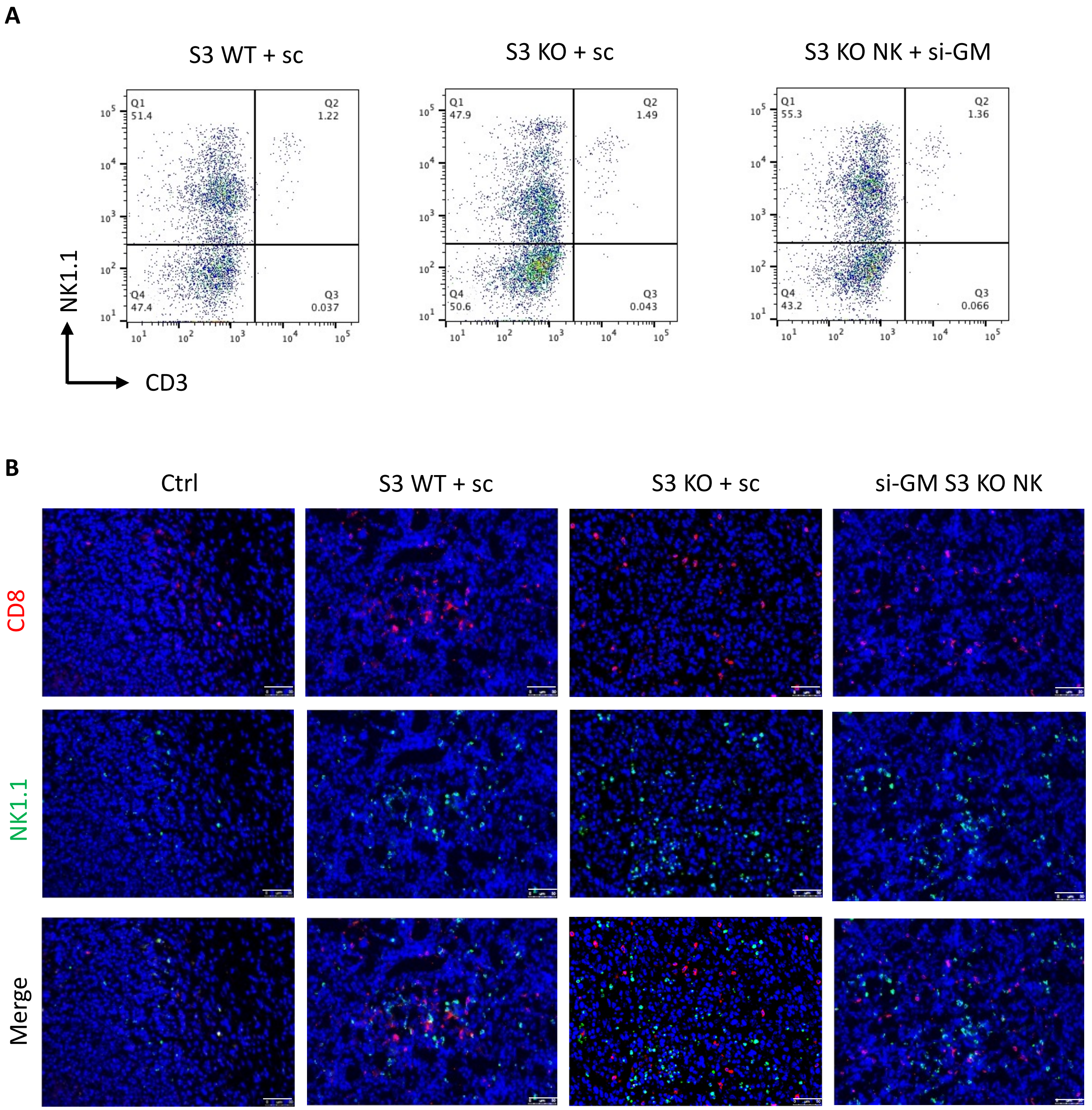

Figure S2. Proportion of NKT cells in BM-NK cells. (A) Flow cytometry analysing the proportion of NKT cells in BM-NK cells used for adoptive cell therapy with NK1.1 and CD3 staining. Results showed that the percentage of NKT cells in NK cells used for adoptive cell therapy in all three groups are less than 2%. **(B)** Immunofluorescence staining showed very few NKT cells were found in tumor microenvironment of mice receiving NK cell therapies. Scale bar, 50 µm.

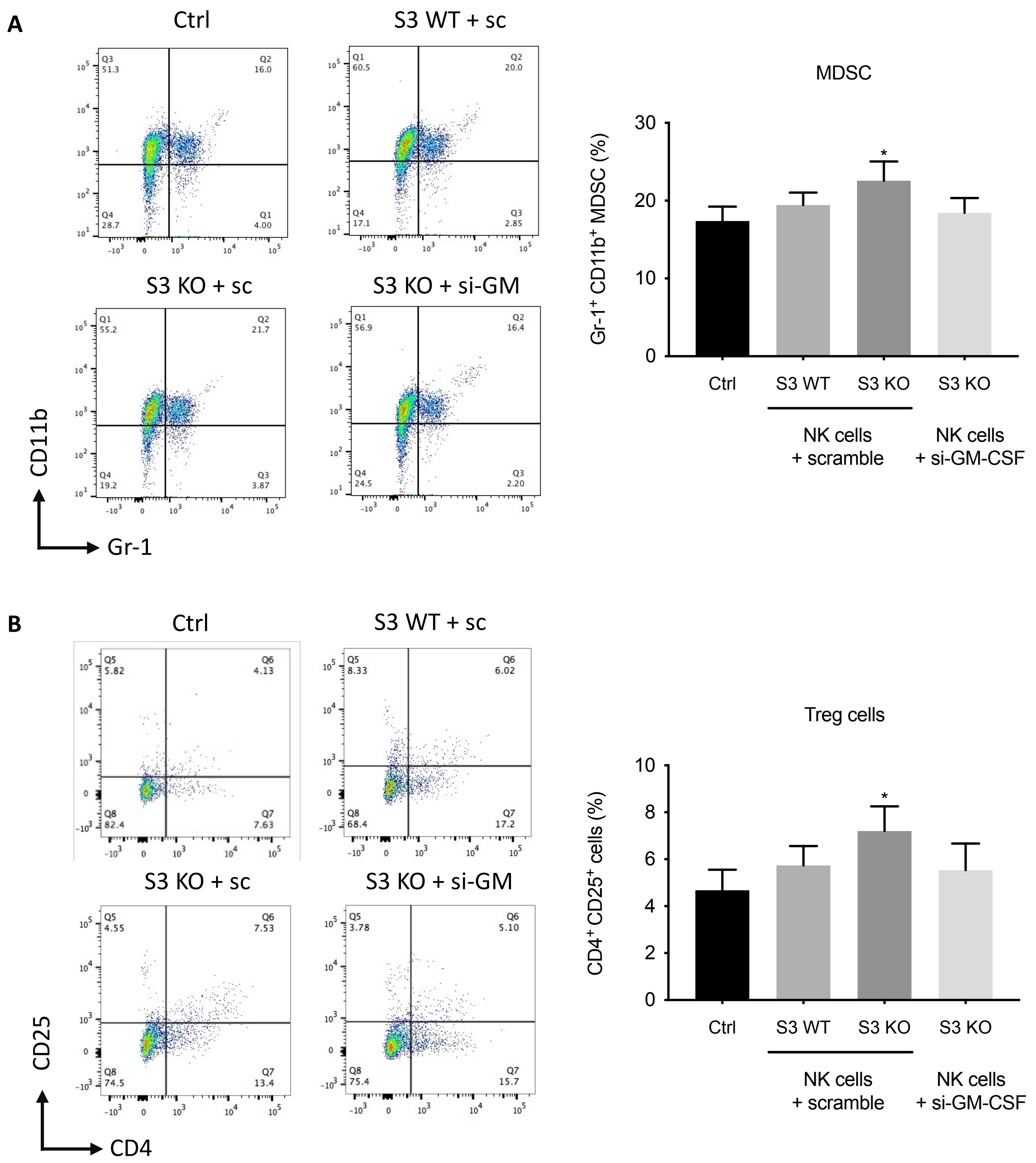

Figure S3. NK-derived GM-CSF slightly enhances the accumulation of Tregs and MDSC in the tumor microenvironment. (A) Flow cytometry analysing the percentage of C11b$^+$ GR-1$^+$ MDSC in the LLC tumor. **(B)** Flow cytometry examining the proportion of CD4$^+$ CD25$^+$ Treg cells in the LLC tumor. Each bar represents the mean ± SD for groups of four to five mice. * $p < 0.05$ compared to Ctrl.c

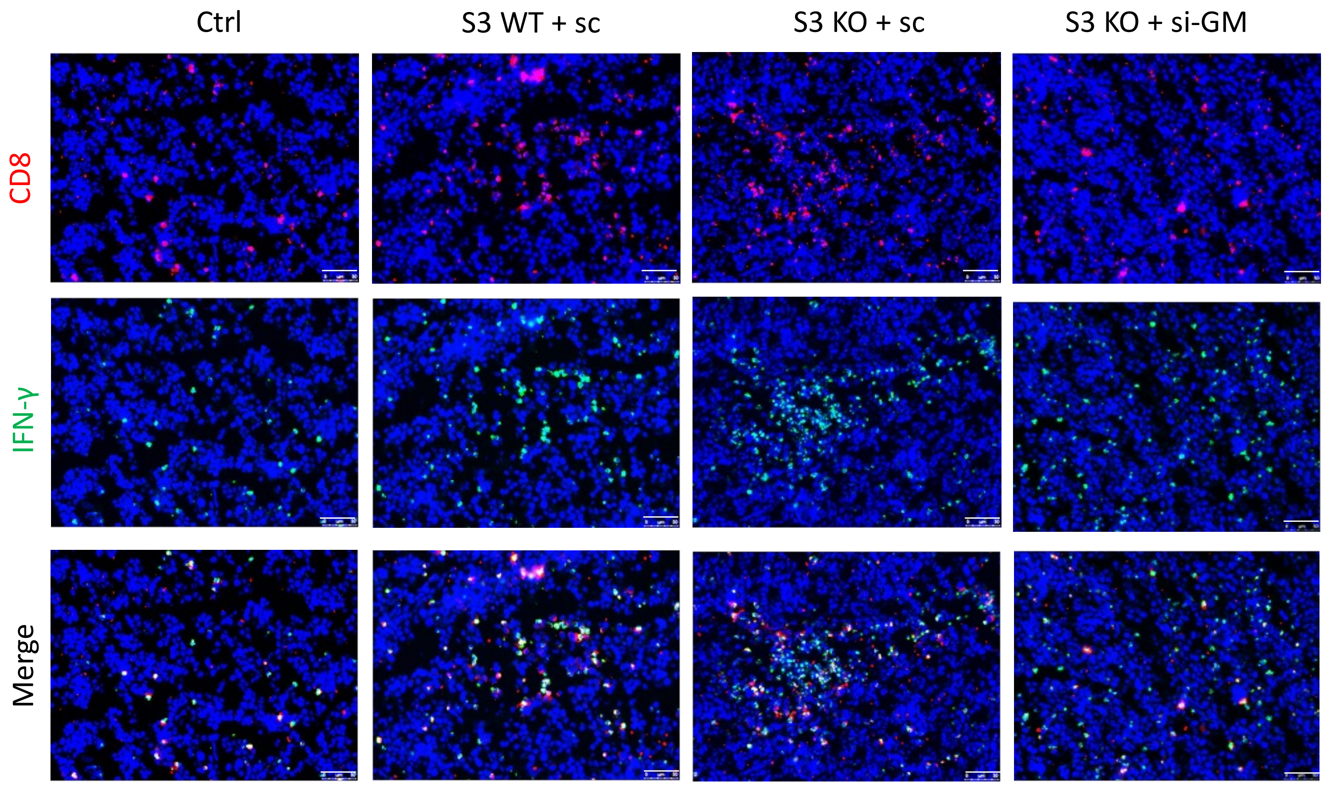

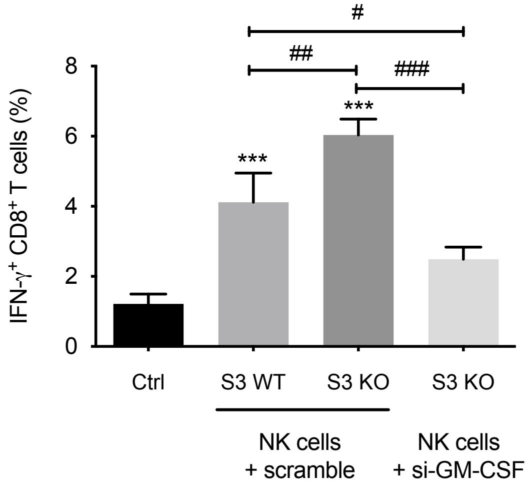

Figure S4. NK-derived GM-CSF promotes CD8+ T cell recruitment in lung carcinoma, which is the major source of IFN-γ in the tumor microenvironment. Immunofluorescence staining detecting of IFN-γ-producing CD8 T cells in LLC tumor microenvironment. Scale bar, 50 µm. Each bar represents the mean ± SD for groups of three mice. ** $p < 0.01$, *** $p < 0.001$ compared to Ctrl; # $p < 0.05$, ## $p < 0.01$, ### $p < 0.001$ as indicated. S3 WT: Smad3 wild-type; S3 KO: Smad3 knockout; sc or scramble: scramble sequence; si-GM-CSF: siRNA targeting GM-CSF.

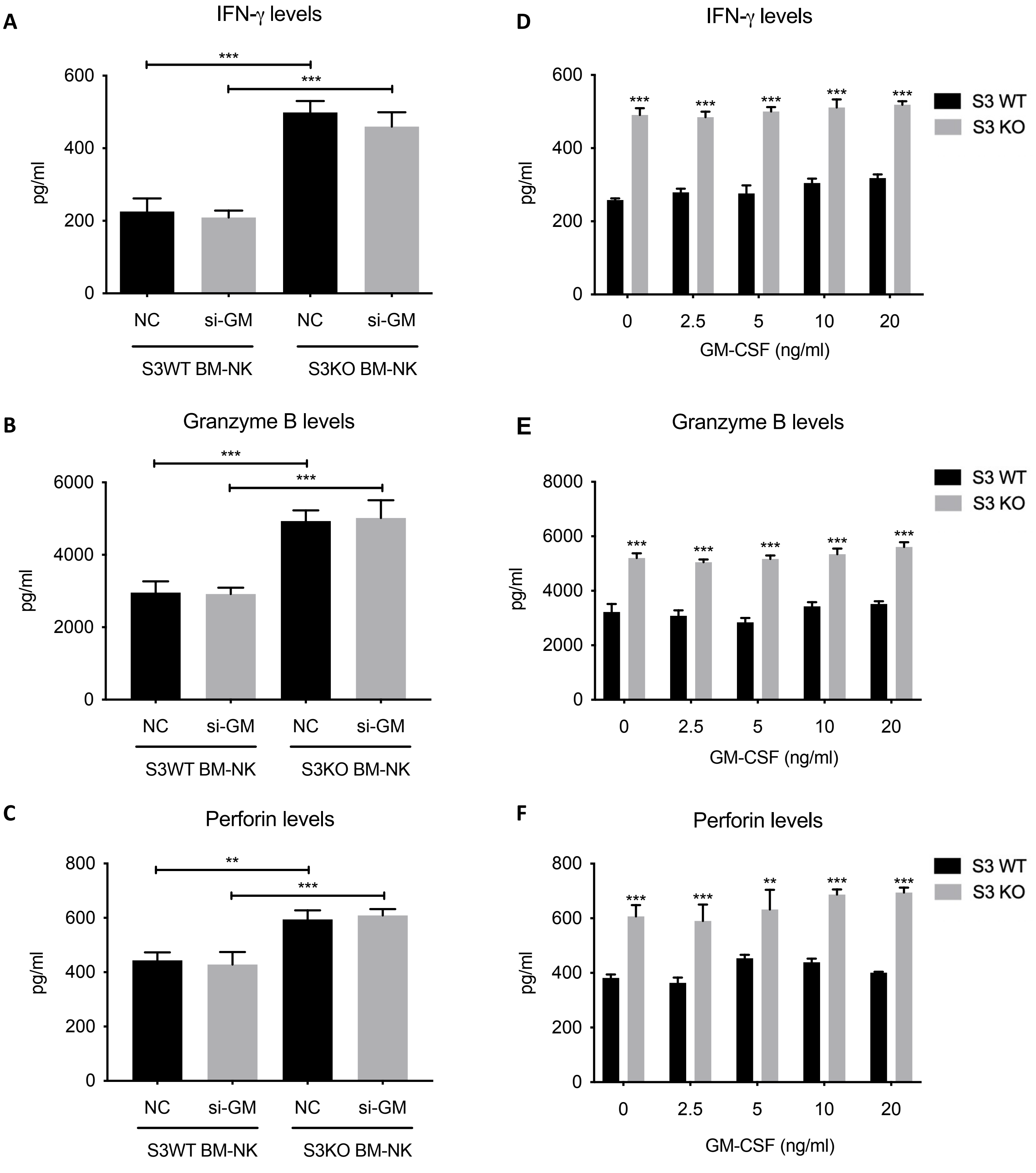

Figure S5. GM-CSF does not influence the productions of anti-cancer cytokines by BM-NK cells in vitro. (A-C) Silencing GM-CSF does not influence cytokine productions by BM-NK cells. ELISA detecting the levels of (A) IFN-γ, (B) granzyme B and (C) perforin in the supernatant of BM-NK cells transfected with either scramble sequence (NC) or siRNA targeting GM-CSF (si-GM) for 24 hours. Each bar represents the mean ± SD for groups of three independent experiments. ** $p < 0.01$, *** $p < 0.001$ as indicated. **(D-F)** Stimulation with recombinant GM-CSF does not influence cytokine productions by BM-NK cells. ELISA detecting the levels of (D) IFN-γ, (E) granzyme B and (F) perforin in supernatant of BM-NK cells treated with recombinant GM-CSF for 24 hours. ** $p < 0.01$, *** $p < 0.001$ compared control (0 ng/ml GM-CSF).
